# Role of free style Libre-Flash Glucose Monitoring: Glycemic control of Type-1 Diabetes

**DOI:** 10.12669/pjms.37.7.4114

**Published:** 2021

**Authors:** Imad A.A. Mohamed, Iman M. Talaat, Hamed A. Alghamdi, Gamal Allam

**Affiliations:** 1 Imad A.A. Mohamed, Department of Microbiology, College of Medicine, Taif University, Taif, Saudi Arabia; 2 Iman M. Talaat, Dept. of Pediatrics, Faculty of Medicine, Ain Shams University, Cairo, Egypt. Diabetes Endocrine Specialist Center, Prince Mansour Military Hospital, Taif, Saudi Arabia; 3 Hamed A. Alghamdi, Academic Medical Center, Prince Mansour Military Hospital, Taif, Saudi Arabia; 4 Gamal Allam, Department of Microbiology, College of Medicine, Taif University, Taif, Saudi Arabia

**Keywords:** Free style libre-flash glucose monitoring, Glycaemic control, Glycated hemoglobin, Type-1 diabetes

## Abstract

**Background & Objective:**

Type-1 diabetics (T1D) usually do not meet guidelines for glycaemic control. This study aimed to determine the benefit of free style libre-flash glucose monitoring system (FSL-FGM) in lowering glycated hemoglobin (HbA1c) in poorly controlled T1D patients.

**Methods:**

This prospective two single arm clinical study included 273 T1D patients, and data collected at one, six and 18 months with concomitant extraction of samples for HbA1c basal and at six and 18 months. The study was conducted in Prince Mansour Military Hospital at Taif, Saudi Arabia from June 2017 to November 2018.

**Results:**

HbA1c % was significantly diminished in patients used FSL-FGM at 6 and 18 months. The median percentage difference in HbA1c at 6 and 18 months versus basal was significantly decreased in those using FSL-FGM. Within diabetics using FSL-FGM, the median difference in HbA1c after 18 months was significantly decreased in patients with HbA1c >10% compared to those with HbA1c <10%. Estimated HbA1c by FSL showed a significant correlation with HbA1C assayed in the blood. The snapshot information showed a highly significant difference in average glucose with low significant difference in hypoglycemia parameters. The FSL-FGM provides significant changes in HbA1c in diabetic patients without observed risk for hypoglycemia.

**Conclusions:**

The dynamic way of blood glucose monitoring using FSL-FGM provides improvement in HbA1c in diabetic patients without observed risk for hypoglycemia.

## INTRODUCTION

The majority (>75%) of children/adolescents with Type-1 diabetes (T1D) failed in achieving optimum glycemic control according to International Society for Pediatric and Adolescent Diabetes/ American Diabetes Association guidelines where glycated haemoglobin (HbA1c) should less than 58 mmol/mol (7.5%).^1^,^2^ The use of HbA1c testing point-of-care can help in treatment changes during time point visit between patients and providers.^3^ However, HbA1c does not give an image about glycemic variability or hypoglycemia. For diabetic patients liable to this variability, ideally glycemic control is to be evaluated by the interpretation of results from both monitoring of blood glucose and HbA1c.^4^

Self-monitoring of blood glucose (SMBG) was considered as an important intervention to illustrate the value of tight glycemic control on long term diabetic complications,^5^ because patients have the opportunity to assess efficiency of their therapy.^6^ However, due to the pain and discomfort of finger sticks and the need to wake up in the night to test blood glucose levels when nighttime hypoglycemia is a concern, it is especially difficult to have frequent self-monitoring in children.^7^

Both, continuous glucose monitoring (CGM) and flash glucose monitoring (FGM), measure interstitial glucose which correlates well with plasma glucose.^4^,^8^ FGM differs from CGM in that information about the person’s glucose levels and trends is available when the sensor is scanned. In comparison, CGM systems monitor send information to a device or display monitor without interruption throughout the day, and can alert a user if glucose levels are outside a pre-set limit.^9^

This study aimed to observe the usefulness of 18-month usage of free style libre-flash glucose monitoring system (FSL-FGM) in pediatric/adolescent diabetics assessing benefit of usage of FSL-FGM in lowering HbA1c, and to correlate between the different variables detected by the FSL-FGM system soft-ware and the changes in HbA1c.

## METHODS

A prospective two single arm clinical study included T1D patients recruited from Diabetes Endocrine Specialty Clinic, Prince Mansour Military Hospital at Taif, Saudi Arabia. Participating patients were followed for 18 months period from June 2017 to November 2018. Patients eligible were diabetics on insulin therapy, poorly controlled with mean HbA1c prior to the study over nine months period >9% (calculated mean of 3 HbA1c tests done three months apart). Guardian of patients signed a written informed consent form, and the study was approved by the Institutional Ethical Committee (TU 38-5788 on 7/05/2018).

All T1D participants have negative celiac screening, average activity level with no diabetic complications and on multiple dose injections regimen. Patients were randomized to participate by either using FSL-FGM (Group-1) or to be controls (Group-2). The FSL-FGM system was adjusted and used according to FSL user manual, and as mentioned previously. 10-12 The supply of FSL-FGM was guaranteed for Group-1 patients for the whole 18 months period of the study. All patients were advised to keep glucose readings within the pre-prandial range of 70-140 mg/dl with upmost postprandial readings below 180 mg/dl using FSL for Group-1 and blood glucose measurements for Group-2. Uploading of FSL-FGM devices was performed for Group-1 patients after 4 weeks, six and 18 months from onset of the study with concomitant extraction of samples for HbA1c at six and 18 months for both groups which was analyzed. Those who were showing problems of repeated detachment of the sensor were excluded from the study.

**Fig. 1:**
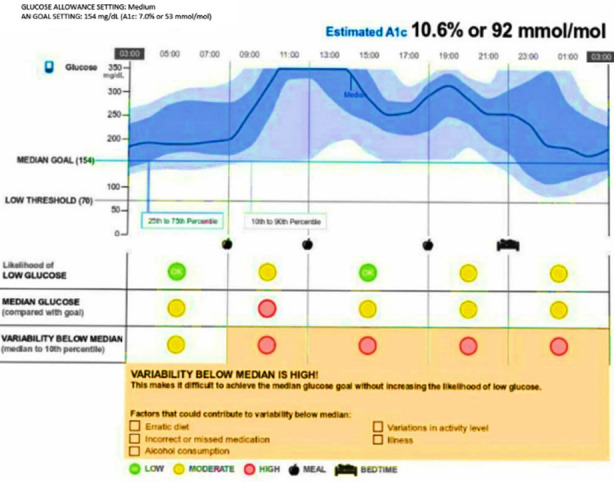
Ambulatory glucose profile summarizes glucose data into percentiles (up) and stoplight chart focusing on potential trouble spots over a period of time (down), adopted from FSL-FGM downloaded data from one of patients included in the study.

The software analyzed the glucose readings at 3 time points: four weeks, six months and 18 months and provided two analytical sheets for each time point as follows:

1. The snapshot sheet provided information about estimated HbA1c, average glucose in mg/dl (with % above target, % in target and % below target), number of low glucose events and average duration, % of sensor data captured, and number of daily scans. This information provided were compared between the two groups of patients as regards three time points: four weeks, six months and 18 months.
2. The glucose pattern insights sheet: the ambulatory glucose profile summarized glucose data into percentiles throughout the day over 3 months period dividing the day into 5-time intervals (Fig.1). The software provided information about each time interval as regards hypoglycemia, median glucose (compared with goal) and variability below median (median to 10^th^ percentile which reflects how difficult to achieve the median glucose goal without increasing the likelihood of low glucose).

A stoplight chart^13^ highlights every time interval as regards the three aforementioned information into green if normal, yellow if moderately abnormal and red if high abnormality detected (Fig.1). The hypoglycemia and variability below median were scored as zero (if green), one (if yellow) and two (if red). The mean of scores obtained from stoplight chart were compared between the two groups of patients in relation to the 3 time points. Detailed analysis for glycemic variability was not done.

Regular assessment of HbA1c every three months was done for the two patients’ groups. Average HbA1c was calculated for each group basal (calculated mean of 3 HbA1c tests done 3 months apart prior to the study) and average HbA1c after 18 months period (calculated mean of 3 HbA1c tests done 3 months apart in the last nine months of the study).

### Statistical Analysis

Data were statistically described in terms of mean ± standard deviation (SD), median and range, or frequencies (number of cases) and percentages when appropriate. Within-group comparison of numerical variables was done using paired t test in comparing the two groups when normally distributed and Wilcoxon signed rank test for paired (matched) samples when not normally distributed. Within the group comparison of numerical variables between more than two time points was done using repeated measures analysis of variance (ANOVA) test through General Linear Model Regression analysis for normally distributed data and using Freidman’s test when data were not normal. Correlation between various variables was done using Pearson moment correlation equation for linear relation in normally distributed variables and Spearman rank correlation equation for non-normal variables/non-linear monotonic relation. All statistical calculations were done using computer program IBM SPSS, version 22 for Microsoft Windows.

## RESULTS

This study included 273 T1D patients, 155 males (57%) and 118 females (43%). The mean age was 11.5±3.76 and the mean duration of diabetes was 5.2±2.6 years. Group-1 (Diabetics using FSL-CGM) included 142 patients and Group-2 (controls) included 131 diabetic patients. Average basal HbA1c percentage was 10.49±1.8 with no significant difference (p > 0.05) between the two groups (Table-I).

**Table I:** Comparison of HbA1c, average HbA1c, median % difference in HbA1c between groups at the 3 time points (basal, 6 months and 18 month) and average hypoglycemia events.

		*Group-1 Diabetics using FSL-FGM*	*Group-2 controls*	*p- value*
Number		142	131	---
Age		12.42±3.89	11.4±4.34	0.042
Sex	Male	84 (59%)	71 (54%)	-----
	Female	58 (41%)	60 (46%)	
Average Basal HbA1c % (mean±SD)		10.56±1.63	10.41±1.99	0.492
Basal HbA1c % (mean±SD)		10.47±1.66	10.52±2.17	0.818
HbA1c % at 6 months (mean±SD)		8.76±1.44	10.42±2.34	0.003
HbA1c % at 18 months (mean±SD)		8.22±1.5	10.24±2.08	0.001
Average HbA1c % after 18 months (mean±SD)		8.47±1.33	10.33±2.04	0.000
Average number of hypoglycemic events over 18 months (mean±SD)		8.24±7.17 Reported by FSL-FGM	14.26±6.7 Reported by blood Glucocheck	0.003
Median % difference in HbA1c %, 6 months versus basal (median)		-7.96	-1.09	0.000
Median % difference in HbA1c %, 18 months versus basal(median)		-8.33	-1.26	0.001
Median % difference in HbA1c %, 18 months versus 6 months (median)		-1.87	-0.99	0.018
Median % difference in average HbA1c %, 18 months versus basal (median)		-9.38	-1.5	0.000

P-value considered significant if < 0.05.

### Effect of using FSL-CGM on HbA1c

There was a significant difference in HbA1c percentage between the two groups after 6 months (p=0.003), 18 months period of time (p=0.001) and average HbA1c after 18 months (p=0.000) (Table-I). Group-1 patients (Diabetics using FSL-CGM) showed a significant decrease (p=0.000) in HbA1c percentage in each time point when comparing basal HbA1c % with corresponding HbA1c % at each time point, whereas controls (Group-2) showed no significant difference (p>0.05) in HbA1c % at the 3 time points (Table-II).

**Table II:** Comparison of HbA1c at the 3 different time points in each group of patients.

	*Group-1 Diabetics using FSL-FGM*			*Group-2 controls*		

	*Mean ±SD*	*Paired T test*		*Mean±SD*	*Paired T test*	
	
		*t- value*	*P -value*		*t -value*	*P- value*
Average Basal HbA1c % (mean±SD)	10.56±1.63	11.30	0.000	10.41±1.99	0.99	0.325
Average HbA1c % after 18 months (mean±SD)	8.47±1.33			10.33±2.04		
Basal HbA1C % (mean±SD)	10.47±1.66	6.86	0.000	10.52±2.17	0.71	0.478
HbA1C % at 6 months (mean±SD)	8.76±1.44			10.42±2.34		
Basal HbA1c % (mean±SD)	10.47±1.66	9.244	0.000	10.52±2.17	1.96	0.052
HbA1C% at 18 months (mean±SD)	8.22±1.5			10.24±2.09		

P value considered significant if < 0.05.

As shown in Table-I, the median percentage difference in HbA1c basal versus 6 months was highly significantly decreased (p=0.000) in Group-1 (-7.96%) compared to Group-2 (-1.09%). Similarly, the median percentage difference in basal HbA1c percentage versus 18 months was significantly reduced (p=0.001) in diabetics using FSL-CGM (-8.33%) compared to the control group (-1.26%). Likewise, the median percentage difference in HbA1c at six months versus 18 months was significantly decreased (p=0.018) in Group-1 (-1.87%) compared to Group-2 (-0.99%). In addition, the median percentage difference in average basal HbA1c versus 18 months was highly significantly reduced (p=0.000) in diabetics using FSL-CGM (-9.38%) compared to controls (-1.5%) (Table-III).

**Table III:** Comparison of median % difference in HbA1c at 18 months versus basal with HbA1c cut off point 10% for comparison in study groups.

	*Group-1 Diabetics using FSL-FGM*			*Group-2 Controls*		

	*HbA1c ≤10*	*HbA1c >10*	*P- value*	*HbA1c ≤10*	*HbA1c >10*	*P- value*
Number	58	84	---	59	72	---
Median % difference in HbA1c ,18 months versus basal (mean±SD)	-5.61±9.37	-12.37±9.12	0.000	2.33±10.76	-2.6±7.93	0.064

P value considered significant if < 0.05.

In this study, diabetic patients were stratified according to cut off point HbA1c of 10%. In diabetics using FSL-CGM, our results revealed that median % difference in HbA1c after 18 months versus basal was highly significantly decreased (p= 0.00) in patients with HbA1c >10% (-12.37±9.12) compared to those with HbA1c ≤10% (-5.61±9.37). However, in controls, there was no significant difference (p=0.064) in the median % difference in HbA1c between the same two time points (Table-II).

For hypoglycemia, number of low glucose events reported by FSL-FGM over 18 months in Group-1 was lower 8.24±7.17calculated by FSL-FGM compared to 14.26±6.71 in Group-2 represented by number of hypoglycemic readings in blood reported in monitoring chart given by each patient (Table-I).

### Analyzing the data of hyperglycemia and euoglycemia obtained by FSL-FGM software in Group-1 diabetic patients

Estimated HbA1c by FSL-software at both 6- and 18-months’ time periods showed a significant correlation with HbA1c assayed in the blood at the same time point (p=0.013) (Table-IV). The snapshot information was compared at the 3 time points with no significant difference between percentage of captured data and number of scans per day (p=0.0769 and 0.358, respectively). The snapshot information by FSL-software showed a highly significant difference in average glucose, % above the target and % within the target (p=0.000 per all) (Table-IV).

**Table IV:** Comparison of the snapshot sheet information by FSL-FGM software at the three time points in Group-1 diabetic patients.

	*Group-1: Diabetics using FSL-FGM*			

*The snapshot information by FSL-FGM software*	*At 4 weeks visit*	*At 6 months visit*	*At 18 months visit*	*p- value*
Average glucose(mean±SD)	255.36±51.77	215.6±41.93	196.14±43.29	0.000
% above target (mean±SD)	77.77±12.35	72.23±12.22	62.86±16.48	0.000
% within target(mean±SD)	15.9±8.27	19.93±9.10	30.39±12.85	0.000
% below target(mean±SD)	6.2±6.10	7.84±6.29	7.15±7.20	0.05
Number of Low glucose events(mean±SD)	7.66±10.00	8.13±8.15	9.12±8.97	0.035
Average hypoglycemia duration in minutes(mean±SD)	79.17±49.96	84.76±49.34	86.55±47.48	0.024
% captured data(mean±SD)	73.43±20.19	75.24±21.12	74.63±22.48	0.769
Number of scans(mean±SD)	9.9±15.74	10.34±9.375	11.69±11.45	0.358

P value considered significant if < 0.05.

The median % difference in HbA1c at 18 months versus 4-week time points was positively correlated with the median % difference in the average glucose (p=0.003), median % difference within the target (p=0.001). However, the median % difference in HbA1c at 18 months versus 4-week time points was negatively correlated with the median % difference in the above target (p=0.002) (Table-V).

**Table V:** Correlation between median % difference in HbA1C 18 months versus 4 weeks in diabetics using FSL-FGM (Group-1) with median % difference of selected data obtained from the snapshot sheet 18 months versus 4-week time points.

*Median % difference in some selected data obtained from snapshot sheet, 18 months versus4 week time points*	*Median % difference in HbA1C 18 months versus 4-week time points*	

	*Pearson correlation*	*P- value*
Median % difference Average Glucose	0.69	0.003
Median % difference above target	-0.75	0.002
Median % difference in target	0.8	0.001
Median % difference below target	0.45	0.04
Median % difference average hypoglycemia	0.55	0.05

P-value considered significant if < 0.05

### Analyzing the data of hypoglycemia obtained by FSL-FGM software in Group-1 diabetic patients

The difference between the 3 time points as regards % below target, number of low glucose events and average hypoglycemia duration in minutes showed low significant difference (p=0.05, 0.035 and 0.026, respectively) (Table-IV). The median % difference in HbA1c at 18 months versus 4-week time points was positively correlated with median % difference below target (p=0.04) and median % difference in average hypoglycemia (p=0.05) (Table-V).

## DISCUSSION

In this study, two groups of diabetic children/adolescents were compared to highlighting the effect of using FSL-FGM in glucose monitoring on both short-term and relatively longer-term glycemic control. Many studies reported the significant reduction in HbA1c in T1D patients being adherent to SMBG^14^,^15^, CGM^16^, or FGM.^17^ Data of the present study revealed statistically significant improvement in HbA1c of diabetic patients using FSL-FGM over six months period compared to diabetics who not using FSL-FGM (p=0.003). This finding is consistent with results of two previous studies conducted over three months 17 and 6 months.^18^

A study performed over 18 months period using a cohort of T1D patients has demonstrated a reduction in HbA1c at six months, but not yet in another study.^18^ This finding is in agreement with our short-term results which showed median % difference in HbA1c at 6 months versus basal equals -7.96 %. In addition, this difference noted to be highly significant between the two groups (p=0.000) and within mean HbA1c in Group-1 between the three time points (p=0.000). However, regarding the long term change, there is a discrepancy between our data and results of Walton-Betancourth and Amin, 2017. 18 We found that with continuous usage of FSL-FGM resulted in a fine adjustment in blood glucose and hence HbA1c. This was evident by the low median % difference at 18 months versus 6 months (-1.87%, p=0.018) as an index of significant difference between the 2 groups and continued increase in median % difference at 18 months versus basal to be -8.33%. Also, with significant difference in average HA1C after 18 months between the 2 groups (p=0.001).

Using FSL-FGM in poorly controlled diabetics with HbA1c >10% showed median % difference -12.37% versus -5.6% in those within the same group with HbA1c ≤10% with significant difference (p=0.000). This result may highlight advantage of using FSL-FGM in glycemic control in very poorly controlled diabetic patients. This also explains the significant difference in HbA1c mean after 6 months between the two groups (p=0.003) as the basal HbA1c mean was 10.47±1.66. In contrast, Al Hayek et al., 2017^17^ reported that the basal HbA1c mean was 8.5±1.07 (p= 0.008), and the study of Walton-Betancourth S et al. and Amin R. et al., 2017^18^ showed the basal HbA1c mean was 7.9±1 (p=0.03). Based on the aforementioned data, we can speculate that as the higher basal HbA1c mean, the greater reduction in HbA1c will be expected. As far as we know, this is the first study to highlight such concern which may be valuable in selecting patients who achieve the highest benefit from FSL-FGM.

The estimated HbA1c provided by the FSL-FGM software (over 3-months period) was positively correlated to HbA1c assayed at the simultaneous 3 months (p=0.013). However, this low significant correlation could be explained by the high MARD of FSL-FGM readings over 14 days usage, compared to capillary blood glucose testing that reaching up to 13.9% in children. But according to what is mentioned in the FSL user manual, the system has a MARD of 9.7% over 10 days without finger-stick calibration. This finding suggests a reduction in the duration of the usage of the sensor to 10 days for better gluco check achievements and hence the software output data, but this may be claimed to be cost ineffective. 19

FSL-FGM captured data in Group-1 showed that the significant drop noted in HbA1c and hence % average glucose (p=0.000) in Group-1 patients, as expected, was due to significant changes in both the data and the median % difference between 18 months versus basal as regards % of captured data above target and captured data % in target (both p=0.000). However, the changes in hypoglycemia parameters (% below target, number of low glucose events and average hypoglycemia duration in minutes) and median % difference 18 months versus 4 weeks time points, had low significant difference which is reassuring and supported by the results of IMPACT study.^20^ Moreover, the clear improvement in HbA1c with a slight increase in number and duration of hypoglycemia events and still the significant difference between number of hypoglycemic events over 18-months period between the two groups, both may be attributed to one of the peculiar features of FSL-FGM which is the “trend arrow” that helps patients or guardians to anticipate hypoglycemia and adopt the appropriate management.

These remarkable changes in HbA1c in patients using FSL-FGM can be attributed to the easy-to-understand graph with a quick summary of glucose history and glucose trend arrow provided with each sensor scan which help patients to manipulate their blood glucose in addition to the ambulatory glucose-profile which provides the endocrinologist a way of assessing glucose levels on a continuous 24-hour basis that shows how day-to-day decisions impact the control of blood glucose 21, a look beyond HbA1c which provides a move to control of diabetes based on a dynamic overview.

### Limitations of the study

The main limitation of this study was the small sample size however, it is still one of the largest pediatric cohorts using FSL-FGM in glucose monitoring on both short-term and longer-term glycemic control for T1D patients in Taif, Saudi Arabia.

## CONCLUSION

In conclusion, the current study shows that the way of blood glucose monitoring using FSL-FGM provides significant changes in HbA1c in diabetic patients without observed risk for hypoglycemia.

### Author`s Contribution:

**IMT and GA** conceived the design of the study and prepared the manuscript. **IMT, IAM, HAA and GA** collecting data and performed the laboratory investigation. All authors approved the manuscript and accountable for the accuracy or integrity of the work.
